# Supplementary material for: COZOID: contact zone identifier for visual analysis of protein-protein interactions
Source: BMC Bioinformatics. 2018 Apr 6;19:125. doi: 10.1186/s12859-018-2113-6 (PMC5889581; doi:10.1186/s12859-018-2113-6)
Supplement: Supplementary file 2 — Software build. Executable binary file of the software tool. (ZIP 118,784 kb) [file 12859_2018_2113_MOESM2_ESM.zip › cozoid/java/docs/beansbinding-1.2.1-doc.zip!/org/jdesktop/swingbinding/JListBinding.DetailBinding.html]

JListBinding.DetailBinding (Beans Binding)


---


|  |  |  |
| --- | --- | --- |
| **Overview** \| **Package** \| **Class** \| **Use** \| **Tree** \| **Deprecated** \| **Index** \| **Help** | |  |
| **PREV CLASS**   **NEXT CLASS** | **FRAMES**    **NO FRAMES**     **All Classes** |  |
| SUMMARY: NESTED \| FIELD \| CONSTR \| METHOD | DETAIL: FIELD \| CONSTR \| METHOD |  |


---


## org.jdesktop.swingbinding Class JListBinding.DetailBinding

```
java.lang.Object
  org.jdesktop.beansbinding.Binding
      org.jdesktop.swingbinding.impl.AbstractColumnBinding
          org.jdesktop.swingbinding.JListBinding.DetailBinding
```

**Enclosing class:**: JListBinding<E,SS,TS>

---

``` public final class JListBinding.DetailBinding extends AbstractColumnBinding ```

`DetailBinding` represents a binding between a property of the elements
in the `JListBinding's` source `List`, and the values shown in
the `JList`. Values in the `JList` are aquired by fetching the
value of the `DetailBinding's` source property for the associated object
in the source `List`.

A `Converter` may be specified on a `DetailBinding`. Specifying a
`Validator` is also possible, but doesn't make sense since `JList`
values aren't editable.

`DetailBindings` are managed by their `JListBinding`. They are not
to be explicitly bound, unbound, added to a `BindingGroup`, or accessed
in a way that is not allowed for a managed binding.

**See Also:**: `JListBinding.setDetailBinding(Property, String)`

---

**Nested Class Summary**

**Nested classes/interfaces inherited from class org.jdesktop.beansbinding.Binding**

`Binding.SyncFailure, Binding.SyncFailureType, Binding.ValueResult<V>`


**Method Summary**

**Methods inherited from class org.jdesktop.swingbinding.impl.AbstractColumnBinding**

`bindImpl, getColumn, setColumn, unbindImpl`

**Methods inherited from class org.jdesktop.beansbinding.Binding**

`addBindingListener, addPropertyChangeListener, addPropertyChangeListener, bind, bindUnmanaged, firePropertyChange, getBindingListeners, getConverter, getName, getPropertyChangeListeners, getPropertyChangeListeners, getSourceNullValue, getSourceObject, getSourceProperty, getSourceUnreadableValue, getSourceValueForTarget, getTargetNullValue, getTargetObject, getTargetProperty, getTargetValueForSource, getValidator, isBound, isManaged, isSourceUnreadableValueSet, notifySynced, notifySyncFailed, paramString, refresh, refreshAndNotify, refreshAndNotifyUnmanaged, refreshUnmanaged, removeBindingListener, removePropertyChangeListener, removePropertyChangeListener, save, saveAndNotify, saveAndNotifyUnmanaged, saveUnmanaged, setConverter, setManaged, setSourceNullValue, setSourceObject, setSourceObjectUnmanaged, setSourceProperty, setSourceUnreadableValue, setTargetNullValue, setTargetObject, setTargetObjectUnmanaged, setTargetProperty, setValidator, sourceChangedImpl, targetChangedImpl, throwIfBound, throwIfManaged, throwIfUnbound, toString, unbind, unbindUnmanaged, unsetSourceUnreadableValue`

**Methods inherited from class java.lang.Object**

`clone, equals, finalize, getClass, hashCode, notify, notifyAll, wait, wait, wait`

---


|  |  |  |
| --- | --- | --- |
| **Overview** \| **Package** \| **Class** \| **Use** \| **Tree** \| **Deprecated** \| **Index** \| **Help** | |  |
| **PREV CLASS**   **NEXT CLASS** | **FRAMES**    **NO FRAMES**     **All Classes** |  |
| SUMMARY: NESTED \| FIELD \| CONSTR \| METHOD | DETAIL: FIELD \| CONSTR \| METHOD |  |


---
